# Linguistic changes in spontaneous speech for detecting Parkinson’s disease using large language models

**DOI:** 10.1371/journal.pdig.0000757

**Published:** 2025-02-10

**Authors:** Jonathan L. Crawford

**Affiliations:** Department of Electrical and Computer Engineering, Boston University, Boston, Massachusetts, United States of America; Brigham and Women’s Hospital, Harvard Medical School, UNITED STATES OF AMERICA

## Abstract

Parkinson’s disease is the second most prevalent neurodegenerative disorder with over ten million active cases worldwide and one million new diagnoses per year. Detecting and subsequently diagnosing the disease is challenging because of symptom heterogeneity with respect to complexity, as well as the type and timing of phenotypic manifestations. Typically, language impairment can present in the prodromal phase and precede motor symptoms suggesting that a linguistic-based approach could serve as a diagnostic method for incipient Parkinson’s disease. Additionally, improved linguistic models may enhance other approaches through fusion techniques. The field of large language models is advancing rapidly, presenting the opportunity to explore the use of these new models for detecting Parkinson’s disease and to improve on current linguistic approaches with high-dimensional representations of linguistics. We evaluate the application of state-of-the-art large language models to detect Parkinson’s disease automatically from spontaneous speech with up to 78% accuracy. We also demonstrate that large language models can be used to predict the severity of PD in a regression task. We further demonstrate that the better performance of large language models is due to their ability to extract more relevant linguistic features and not due to increased dimensionality of the feature space.

## Author summary

Our work explores the use of large language models to detect Parkinson’s disease from spontaneous speech. Parkinson’s disease is the second most common neurodegenerative disorder, affecting over 10 million people worldwide. However, it is often diagnosed late due to the complex nature of symptoms and the limitations of existing detection methods. Language impairment can precede the physical symptoms currently used for diagnosis, which suggests speech analysis could allow for earlier detection. We evaluate several state-of-the-art large language models to automatically analyze transcripts of spontaneous speech from Parkinson’s patients and healthy controls. The large language models extract linguistic features such as word usage, grammar, meaning, and context into high-dimensional representations. We demonstrate that the models can be used to detect Parkinson’s disease and outperform prior methods. This demonstrates the potential for artificial intelligence to aid in screening for Parkinson’s disease based on subtle speech patterns. The work paves the way for further research into leveraging these powerful language models as affordable, non-invasive screening tools. Ultimately, the use of large language models could allow for earlier diagnosis and treatment intervention, improving quality of life for millions affected by Parkinson’s disease.

## Introduction

Parkinson’s disease (PD) is the second most prevalent neurodegenerative disorder with over ten million active cases worldwide, one million new diagnoses per year, and an exponentially growing incidence rate [1]. The global prevalence of PD continues to rise due to increased life expectancy and industrialization [2]. PD is a chronic and progressive neurodegenerative disorder that induces physical and cognitive impairment. The pathogenesis and pathophysiology of the disease are poorly understood. Current research indicates that the pathogenesis of PD involves an interplay of unknown genetic susceptibilities and environmental exposures [3]. PD is characterized by the progressive loss of dopaminergic neurons in the substantia nigra and the presence of Lewy bodies, leading to central nervous system degradations. The disease is also characterized by motor symptoms, namely bradykinesia, resting tremor, rigidity, and postural instability. Non-motor symptoms also manifest heterogeneously [4].

PD is clinically diagnosed late relative to the pathogenesis and with low accuracy [5,6]. This can be attributed to multiple confounding factors including the absence of early-stage biomarkers and screening methods, the complex symptomatology of the disease, and the limitations of diagnostic methods in timely detection and differentiation. The pathogenesis of the disease is estimated to begin decades before the manifestation of the phenotypic symptoms necessary for clinical diagnosis [7]. PD is currently diagnosed with clinical evaluations. The clinical evaluation utilizes phenotypic symptoms augmented with neuroimaging to exclude other conditions. The primary symptoms used for PD diagnosis are tremor, rigidity, bradykinesia, and postural instability. These fine motor-skill deteriorations are considered the cardinal and first observable signs of PD. Dependence on these symptoms is problematic given their variability, non-specificity, and potential overlap with other diseases [8]. Inconsistent symptom onset and presentations across populations further add to the complex symptomatology. Reliance on these physical symptoms leads to late detection because these symptoms do not present until around 80% neural degradation [9].

There is a need for additional biomarkers and new methods to detect PD. Language impairment can present in the prodromal phase and precede motor symptoms suggesting that a linguistic-based approach could serve as a diagnostic method for incipient PD [10]. Linguistics models may also be used to detect PD across all stages and enhance other approaches through fusion techniques.

The architecture, parameter structure, and training of the large language models allow the models to extract and encode into text embeddings a unique linguistic feature space representing the morphology, syntax, semantics, and pragmatics of the spontaneous speech signals [11]. The architecture of a large language model is defined by the transformers, attention mechanisms, and depth of layers, along with specific parameter configurations and the nature of its training data. The differing architecture of each model results in embeddings that capture distinct linguistic patterns. Specific dimensions in this space include the usage of syntactic structures, the frequency of certain words or phrases, the presence of semantic themes, the distribution of parts of speech, and the occurrence of named entities [12]. For PD detection, language impairments may align with dimensions in the feature space including semantic richness, grammatical usage, fluency patterns, and syntactic complexity. For instance, reduced information content may appear as low semantic richness, impaired grammaticality may be evident in deviations from standard grammar, disrupted fluency may be characterized by pauses and self-corrections, and reduced syntactic complexity may be reflected in simpler sentence structures. Mapping these deficits may allow the models to detect deviations from typical language patterns associated with PD.

For example, Bidirectional Encoder Representations from Transformers (BERT) has been used to detect PD [13]. The field of large language models is advancing rapidly, which presents the opportunity to explore the use of the new models for detecting PD and to improve on current linguistic approaches with high-dimensional representations of linguistics [11].

The purpose of this work is to demonstrate the potential of state-of-the-art large language models to improve the field of detection of PD using linguistics. This may eventually lead to a detection method for PD in the prodromal phase. We evaluate the application of state-of-the-art large language models to detect PD from spontaneous speech. The state-of-the-art large language models lead to improved performance over the prior methods using our implementation. We also demonstrate that large language models can be used to predict the severity of PD. We expect that our work will be built upon by us and future researchers to eventually develop a method to detect early onset PD in clinical applications.

## Results

**Fig 1:**
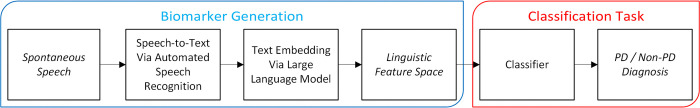
A high-level representation of the methodology. Italicized boxes signify inputs and outputs.

We report on comparing multiple methods to detect PD using state-of-the-art large language models. A high-level description of a system to detect PD is detailed in Fig 1. The system inputs digitized spontaneous speech from participants. The speech either belongs to a control group without PD or a group that has PD of varying degrees of severity. The speech is transcribed automatically with an automated speech recognition model. A large language model then generates a linguistic feature space from the transcription. A classification algorithm subsequently processes the feature space to make a PD/Non-PD diagnosis. Low-level details on each step are presented below. The computer code used to create the results shown in this paper is available upon request from the author.

### Biomarker generation

#### Speech-to-text via automated speech recognition

The audio files are automatically transcribed into textual form using Whisper, an automated, multilingual speech recognition model from Open AI [14]. The translation endpoint is used to transcribe the audio into English.

#### Text embeddings via large language models

Text embeddings are generated from the transcriptions using large language models. We evaluate the efficacy of multiple state-of-the-art large language models. Each model generates a high dimensional linguistic feature space with each dimension representing different linguistic features determined by the architecture and training of the models. The models were chosen because they are widely applied and considered to be state-of-the-art as of the writing of this paper.

We present results from: Bidirectional Encoder Representations from Transformers (BERT) [15]; XLNet [16]; Generative Pre-trained Transformer 2 (GPT-2) [17]; text-embedding-ada-002 [18,19]; and text-embedding-3-small and text-embedding-3-large [20]. We note that BERT has been previously applied for PD detection [13]. We also implement Word2Vec [21], which is a word embedding technique, for comparison with prior linguistic methods.

The dimensionality of the text-embedding-3-small and text-embedding-3-large outputs can be reduced through an application programming interface (API) endpoint parameter. This represents a trade-off between performance and the cost of using embeddings. Specifically, embeddings are shortened internally by the model without losing their concept-representing properties [22]. We present results from the two models at both their default (maximum) dimensionality and at a reduced dimensionality, adjusted to match that of the other evaluated models. This approach enables a direct comparison of performance, standardized based on dimensionality, between these models and the other models evaluated.

### Classification

The classification task presents challenges due to the high dimensionality of the feature spaces compared to the small size of the dataset. A support vector machine (SVM) is utilized for its robustness when handling such high-dimensional data and for its effectiveness in classification tasks through the creation of optimal hyperplanes in a transformed feature space. Specifically, SVM models are resistant to overfitting when regularized [23]. The embeddings are standardized to allow the SVM model to process the feature space [24]. All dimensions of the embeddings are used to train the SVM.

Assessing performance is challenging due to the limited dataset sizes. To address this, 10-fold stratified cross-validation is employed to tune hyperparameters and provide a stochastic estimate of performance. We follow the procedure of [25] to allow comparison of results with others in the field who also employ this standardized procedure. The dataset consists of 100 samples, so 90 are used as the training set and 10 are treated as an unseen and validation set in each fold. A grid search is used to optimize the hyperparameters based on accuracy. The hyperparameters considered are the kernel type, the regularization parameter (C), and the kernel coefficient gamma. The kernel types considered are Polynomial, Radial Basis, and Sigmoid functions, which are denoted in the results section below as poly, rbf, and sig, respectively. The regularization parameter ranges over the values [10^−5^, 10^−4^, …,10^5^], which helps in controlling the trade-off between achieving a low training error and a low testing error, thereby avoiding overfitting. The kernel coefficient, which influences the decision boundary, also spans across the same range of values: [10^−5^, 10^−4^, …, 10^5^].

After each fold in the cross-validation process, the evaluation metrics are recorded, and their mean values are calculated upon completion of all folds. The evaluation metrics are accuracy, precision, recall, and the area under the curve (AUC). Positives are defined as the group with PD and negatives are defined as the healthy control group. Accuracy is defined as correct predictions over all predictions. Precision is defined as true positives over true positives plus false positives (positive predictions). Recall is defined as true positives over true positives plus false negatives (all positives). Area under the curve is defined as area under the receiver operating characteristic curve, where a perfect area has a value of one. Standard Error is defined as the standard deviation of the metric across folds divided by the square root of the number of samples, ten in this case.

The results of our methods are reported in Table 1.

**Table 1:** Results of the application of state-of-the-art large language models. Performance metrics and standard errors are reported as percentages, expressed as mean (μ) ± standard error (SE). AUC is reported as a fraction.

Model	Dimen-sion	Accuracy	Precision	Recall	AUC	Kernel	C	Gamma
Word2Vec	300	54 ± 4.0	58 ± 5.9	60 ± 6.7	0.44 ± 0.05	linear	10^−3^	10^−4^
BERT	768	66 ± 3.7	64 ± 3.0	80 ± 6.0	0.69 ± 0.06	linear	10^−4^	10^−4^
XLNet	768	61 ± 4.1	65 ± 6.9	56 ± 7.7	0.61 ± 0.03	linear	10^−4^	10^−4^
GPT-2	768	70 ± 4.9	73 ± 5.0	72 ± 8.0	0.76 ± 0.06	linear	10^−3^	10^−4^
text-embedding-ada-002	1536	70 ± 4.7	72 ± 4.4	70 ± 6.8	0.70 ± 0.06	sig	10^3^	10^−3^
text-embedding-3-small	1536	78 ± 3.5	80 ± 4.8	80 ± 7.3	0.78 ± 0.05	sig	10^1^	10^2^
text-embedding-3-small	768	76 ± 4.3	85 ± 5.3	68 ± 7.4	0.80 ± 0.04	sig	10^1^	10^−2^
text-embedding-3-large	3072	73 ± 3.8	72 ± 5.5	80 ± 6.0	0.75 ± 0.05	linear	10^−2^	10^−4^
text-embedding-3-large	768	71 ± 4.8	74 ± 6.0	70 ± 5.4	0.74 ± 0.06	linear	10^−2^	10^−4^

In addition to the classification task, a regression model is developed to predict Movement Disorder Society-Unified Parkinson’s Disease Rating Scale Part III (MDS-UPDRS-III, hereinafter denoted UPDRS) scores using the embeddings. This can be interpreted as predicting the severity of the disease based on the speech samples of the PD group. A Support Vector Regression (SVR) model is selected for its ability to manage high-dimensional data and prevent overfitting through regularization. The embeddings are standardized [24]. Samples without UPDRS scores, representing healthy controls, are excluded from the analysis. The grid search from the classification task is used to optimize the SVR hyperparameters. Leave-One-Out Cross-Validation (LOOCV) is used to tune the hyperparameters and evaluate the performance of the model. The hyperparameters are determined based on the negative mean squared error. LOOCV is then performed to calculate the Root Mean Squared Error (RMSE) for each fold and the mean values and standard errors are reported in Table 2.

**Table 2:** Results of the Support Vector Regression for predicting UPDRS scores. Metrics are Root Mean Squared Error (RMSE) with standard errors. We note that the MFCC+EGEMAPS (acoustic) method uses 10-fold cross validation [26].

Model	Dimension	RMSE	Kernel	C	Gamma
Word2Vec	300	14.5 ± 1.6	sig	10^1^	10^−2^
BERT	768	13.5 ± 1.77	sig	10^2^	10^−3^
XLNet	768	14.6 ± 1.65	sig	10^1^	10^−2^
GPT-2	768	14.3 ± 1.64	sig	10^−3^	10^−4^
text-embedding-ada-002	1536	14.7 ± 1.58	sig	10^2^	10^−2^
text-embedding-3-small	1536	15.3 ± 1.57	sig	10^−3^	10^−4^
text-embedding-3-small	768	14.2 ± 1.47	sig	10^1^	10^3^
text-embedding-3-large	3072	13.4 ± 1.62	sig	10^1^	10^−2^
text-embedding-3-large	768	12.9 ± 1.68	sig	10^1^	10^−1^
MFCC+EGEMAPS (acoustic) [26]	N/A	16.4	N/A	N/A	N/A

## Discussion

We demonstrate that the state-of-the-art large language models can detect PD with up to 78% accuracy using a linguistic feature space generated with large language models. We show that the text-embedding-3 models outperform the other models. This finding is consistent with the benchmarked performance of all of the models across a variety of tasks [27]. The previous research for PD detection with large language models, specifically BERT, is only 66% accurate with our implementation and with the dataset that we used [13]. We demonstrate that the text-embedding-3 models surpass BERT across performance benchmarks.

The performance metrics for text-embedding-3 are largely independent of the dimensionality of the embedding output. In particular, even with the dimensionality reduced to 768 to match that of BERT and of the other large language models, the performance metrics remain better than the other large language models. We conclude that the better performance of large language models is due to their ability to extract more relevant linguistic features and not due to increased dimensionality of the feature space. This counters the notion that higher-dimensional output, with more features, automatically leads to better feature representation and performance metrics. We show that text-embedding-3 models with the same dimensionality as other LLMs outperform these models. This indicates that the architecture of the text-embedding-3 models captures a better representation of the linguistic differences between the control and PD groups, rather than merely outputting more features. The architecture of a large language model refers to the design, engineering, and organization of the model’s layers, structures, and mechanisms, which define how it processes and represents the information.

Additionally, we evaluate the performance of the models using precision and recall metrics, which are algebraically related to false positive and false negative rates. Our best results are a precision of 80% and a recall of 80%. These values are approximately ten points better than the prior art. While we are not clinicians, we surmise that 80% is insufficient for clinical applications. High recall is important to detect as many actual PD cases as possible and reduce missed cases (false negatives). In contrast, high precision is important to avoid false positives and minimize unnecessary follow-up actions. Our precision and recall scores show potential, but achieving near-perfect metrics is essential for these models to move from research to clinical use. The purpose of this paper is to demonstrate the potential of large language models to improve linguistic-based detection. We subjectively feel that a six-point increase in accuracy with our implementation and a ten-point increase in precision and recall merits reporting. This statement is based on the exponential difficulty in improving the accuracy (and area under the ROC) as 100% precision and 100% recall are approached [28].

The regression analysis shows that the linguistic feature spaces generated by large language models can predict the severity of Parkinson’s disease, as quantified by UPDRS scores. Notably, the text-embedding-3 method with a reduced dimensionality of 768 achieves the lowest Root Mean Squared Error (RMSE) of 12.9 ± 1.68. We note that the error bars between the large language models overlap and there may not be a statistical difference between the performances. However, the performance of all the models indicates that the embeddings capture linguistic patterns for all levels of disease severity. The text-embedding-3 method outperforms the results of acoustic methods such as MFCC+EGEMAPS within experimental uncertainty [29]. We note that MFCC+EGEMAPS uses 10-fold cross validation whereas we use LOOCV. LOOCV generally exhibits lower bias but higher variance, while 10-fold CV has higher bias but lower variance [30,31]. Despite these differences, both methods estimate the same underlying out-of-sample performance [30,31]. As a result, we can still compare overall trends. However, absolute values should be interpreted with caution due to the distinct bias–variance trade-offs. The findings suggest that large language models and linguistics can serve as a method for monitoring disease progression and assessing the effectiveness of therapeutic interventions.

We note that the presentation of PD symptoms across populations is not homogenous. The heterogeneity of PD symptoms may limit the ability of detection methods to generalize to all patients and presentations of PD. Dysarthria (acoustic impairment) is estimated to occur in 90% of PD patients [32]. The percentage of PD patients who have language impairment is a subject of ongoing research, and the etiology of language deficits in PD is not definitive [33,34]. Research has hypothesized and correlated cognitive impairment to language impairment in PD, and has estimated cognitive impairment to occur in 80–100% of cases of PD [35]. Language impairments in PD have been characterized by reduced information content, impaired grammaticality, disrupted fluency, and reduced syntactic complexity [33]. For example, individuals with PD produce language that contains fewer correct information units, more grammatical errors, and less complex syntactic structures compared to healthy adults. They may also exhibit difficulties in generating verbs, producing longer sentences due to listing events, and including more pauses and hesitations in speech.

The processes for distilling and interpreting dimensions within the feature spaces generated by large language models are not yet fully understood and are the subject of ongoing research [36]. Specific language impairments in PD may be correlated to specific dimensions in the linguistic feature space produced by a large language model [37–39]. Reduced information content may be captured by analyzing semantic richness and coherence within the text. Impaired grammaticality may be detected through features representing grammatical usage and syntactic correctness, which identify deviations from standard grammar rules. Disrupted fluency may be reflected in features that capture fluency patterns including the frequency and duration of pauses, hesitations, and self-corrections. Reduced syntactic complexity may be observed by analyzing sentence structures and the use of complex grammatical constructions, noting a preference for simpler sentences. Mapping these deficits to corresponding dimensions in the linguistic feature space enables large language models to identify deviations from typical language patterns associated with PD.

Comparison between different detection methods is difficult due to the use of proprietary datasets, differing and insufficient implementation details, differing validation methods, and differing classification algorithms. We aim to overcome these limitations by using a dataset that is in the public domain, providing implementation details, and making our source code available upon request. This enables other researchers to replicate our methods and implementation.

### Past work

Motor-speech impairment is estimated to occur in over 90% of PD cases [40]. Past research has shown that acoustic features extracted from speech signals including prosodic, vocal, and lexical elements can be used to detect PD [41]. Research has also been conducted on the processing of acoustic speech signals for detecting Alzheimer’s disease [42].

Acoustic models of speech have demonstrated high accuracy on performance tasks [43]. However, acoustic features are phonetic and arise from physical changes in the vocal tract [44]. They therefore may not manifest until late into the pathogenesis, which limits the utility of acoustic models for screening and asymptomatic detection [45]. The results from the regression task demonstrates the strengths of LLMs over acoustic features for PD screening, as they can more reliably predict disease severity across different stages of the disease and thus support earlier detection.

Linguistic models have also been proposed for both PD and Alzheimer’s disease. Large language models have been utilized to distinguish Alzheimer’s disease from spontaneous speech with 80.3% accuracy [12]. However, the research is conducted in the context of an aphasia exam, and Alzheimer’s disease and PD have different manifestations of language impairment. BERT has been implemented to detect PD from spontaneous speech [13]. A framework for automated semantic analyses of action stories capturing action-concept markers was developed to distinguish PD [46]. Morphological analysis tools were used to study cognitive impairment and utterance alterations in PD on a Japanese dataset [47]. The research showed that cognitively unimpaired PD patients exhibited different usage rates of morphological language components when compared to the healthy control group. Morphological analysis tools encompass only one of the five linguistic components (morphology), whereas text embeddings capture four, including morphology, syntax, semantics, and pragmatics.

Research reports accuracy of up to 72% accuracy with the PC-GITA dataset using Word2Vec [21] word embeddings on a manual transcription of the PD monologues [48]. Word embedding models are context-independent, meaning each word has the same embedding regardless of its context in a sentence. Therefore, word embeddings primarily extract features at the word level. The study also eliminates punctuation and stop words and performs lexicon normalization. This process results in the loss of linguistic information beyond the semantic component of each isolated word [49]. Manual transcription requires domain- and task-specific knowledge from the transcriber. The transcriber and their domain-experience therefore become a variable in the experiment and may introduce implicit bias during the transcription process [50]. In contrast, automatic speech recognition models do not introduce the variable of the transcriber and provide a uniform and scalable approach. However, automatic speech recognition models may struggle with domain-specific jargon, accents, and speech nuances. Specifically, they may fail to identify, either by commission or omission, the explicit incorrect use of language that may be present in individuals with language impairments. Automated transcription may lower accuracy in performance tasks, specifically in the classification of neurodegenerative diseases [51]. We report the performance metrics of our text embeddings approach, applied to an automatically transcribed version of the monologues. We suspect that the accuracy does not fully represent the capabilities of the approach. If the embedding models were applied to a manually transcribed version of the monologues, we may achieve even better performance.

A summary of results of other methods to detect PD is shown in Table 3. We note that while acoustic methods provide the highest accuracy, they may be limited in application and for detecting PD in the prodromal stage.

**Table 3:** Performance results of comparable studies to detect PD. All studies listed use 10-fold cross validation.

Ref.	Features Generation	Dataset	Classifier	Accuracy
[29]	Mel spectrograms (acoustic)	PC-GITA	2D-CNNs & LSTM	98.6
[48]	Bag of Words	PC-GITA	Random Forest	70.0
[48]	Term Frequency-Inverse Document Frequency	PC-GITA	Random Forest	67.0
[48]	Word2Vec	PC-GITA	SVM	72.0
[13]	Wav2Vec 2.0	See ref.	SVM	69.7
			CNN	73.8
[13]	BERT	See ref.	SVM	61.8
			CNN	74.2
[13]	BETO [52]	See ref.	SVM	62.5
			CNN	77.9

### Limitations

The dataset for training and testing the classifier is small relative to the high dimensionality of the feature spaces, which may restrict the generalizability and scalability of the results. The small dataset size also increases the risk of overtraining the model and makes it challenging to create representative validation and testing sets. However, the Support Vector Machine with regularization may prevent overtraining. Additionally, the small number of samples in test sets limits the implementation of statistical methods to assess significance. We note that our cross-validation strategy may provide an overly optimistic estimate of the performance of the models. This is because the hyper-parameters were optimized on the test set. Nested cross-validation can address this issue. However, we present results using a non-nested cross-validation strategy to allow benchmarking with similar studies in the field with the same validation. We note that text-embedding-3-small outperforms text-emebedding-3-large across performance metrics in our use case. This contradicts the results of standardized performance benchmarks, which show that text-embedding-3-large outperforms text-embedding-3-small [20]. We note that there is a risk of larger LLMs overfitting with a small dataset. We surmise that the performance of the LLMs will improve with more data given the large dimensional output and large internal architecture of the models [12].

The dataset for training and testing the classifier may have the following limitations. The dataset may not be representative of all ethnic diversities and cultures, which may limit scalability of the model [53]. The potential for misdiagnosis in the PD patient group and undiagnosed neurological conditions in the control group introduces uncontrolled variables that could impact the findings of the study. The spontaneous speech data may have biases related to language dependence, dialect, and accent [54]. The speech is of only a single language, which may limit the ability of the model to generalize to other languages. For generating the monologues, participants were asked to describe their daily routine. The nature of the prompt may not induce the participants to exhibit all forms of language impairment present.

The time since disease diagnosis (time post diagnosis) for PD patients ranges from 0.4 to 43 years. The high variability in disease duration may lower the application of the method for early disease detection. However, we note that the mean average time post diagnosis for the 50 PD patients is 11.2 ± 9.9 years. This indicates a skew towards the earlier phase of the disease. Additionally, 46% of the UPDRS scores within the PD cohort fall below the 32-point threshold [55]. This indicates only mild motor impairment. Of all the scores, 76% are beneath the 59-point threshold for severe motor impairment. We also note that 70% of the PD group have H&Y scores below two. A H&Y score below two corresponds to the early stage of PD [56]. The distribution of UPDRS and H&Y scores indicate that the majority of the cohort exhibits only mild to moderate symptoms and are in the early stage of PD.

The publication of "Attention is All You Need" [57] and the release of OpenAI’s GPT-3 model has led to rapid growth in the fields of generative AI and large language models. Large language models may be implemented in a clinical setting to help detect PD in the future. However, this raises several concerns and limitations. One concern is the implementation of a test based on a non-transparent and non-open-source model. There are issues of interpretability when relying on a decision made by a black box in a clinical setting, especially when the model is not 100% accurate. The result of a misdiagnosis may be catastrophic, which raises the question of who is accountable for the decision of the model. Research has shown that large language models may reflect societal biases [58]. These biases can include those related to gender and race. Large language models may perpetuate biases present in their training data. They may also suffer from a lack of training data for specific demographics. In our case, large language models may not have sufficient training data on certain vernaculars and cultures. This lack of data may lead to misdiagnosis. These concerns highlight a larger issue as large language models continue to be implemented in the healthcare field and society [59]. More research is needed to ensure that the models and tests are developed and implemented responsibly.

### Future directions

We recommend that future data collection efforts consider various forms of participant prompting. Examples of different prompts include memory-dependent tasks, narrative construction, abstract thinking, and problem-solving scenarios. Additionally, we suggest considering different mediums for conversational tasks, including monologue, dialogue, and multilogue formats. We recommend that future data collection incorporate a dimension of time. By tracking patients longitudinally, research could capture the progression of the disease and its linguistic markers. This could offer information on how early these markers appear and how they evolve. We also recommend the procurement of a standardized dataset with multiple neurodegenerative disorders and languages.

This research highlights spontaneous speech as a classifiable biomarker through linguistic representation in text embeddings. Based on this observation, several questions and future directions emerge. Previous research has demonstrated high accuracy in using speech signals to distinguish various neurodegenerative diseases [60]. However, most of the research uses healthy controls and positives of the disease for binary classification. It remains unclear whether each neurodegenerative disease has a unique signature in its speech patterns, whether acoustic or linguistic. This raises the question of how these models will perform in the real world, where individuals may have other conditions with similar or overlapping symptoms. This also raises the question of whether a single model, either binary or multimodal, can distinguish between multiple neurodegenerative diseases or diseases with similar presentations.

The manifestation of language impairments across various languages is still unclear. The Spanish-based model may be extended and applied to other languages. Techniques such as transfer learning and zero-shot learning may offer effective adaptation strategies for new languages. There also remains the possibility of increasing the accuracy and performance of our approach in this classification task by utilizing more complex classification methods such as computational Neural Networks and Deep learning. Long Short-Term Memory (LSTM) and Recurrent Neural Networks (RNN) are considered effective models for distilling and classifying text embedding feature spaces and may be applied to increase accuracy [61]. Feature spaces may be fused together to improve the accuracy and robustness of a classification method if the features are orthogonal [62]. Acoustic and linguistic impairments stem from different pathophysiological mechanisms and therefore may be orthogonal. Therefore, a fusion method incorporating acoustic and linguistic features may also be developed to increase performance [63].

There has been extensive research in developing new biomarkers and detection methods for PD [41,64,65]. Recent advances include the identification of blood-based protein markers through proteomic phenotyping, progress in neuroimaging techniques for assessing dopamine system function, recognition of REM sleep behavior disorder as a preclinical marker, the use of wearable devices and smartphones for collecting digital biomarkers, and the application of artificial intelligence to analyze nocturnal respiratory signals for PD assessment in home settings [41,65,65]. Linguistic features may be combined with these methods to enhance overall detection accuracy and provide a more robust view of disease markers.

## Materials and methods

### Dataset (spontaneous speech)

The PC-GITA dataset is used in this study [66]. The dataset consists of the spontaneous speech of 50 subjects with PD and 50 health controls (HC) for a total of 100 samples in the dataset. The data are age and gender matched. The p-value for age, calculated with a two-sided Mann-Whitney U test, is 0.99. The data consist of monologues where each subject is asked to speak about what they do on a normal day. The average duration of the monologues is 44.86 seconds. The speech is of native Colombian Spanish speakers. The recordings were taken with the PD patients in the ON state, which refers to a period of time when medication effectively alleviates the motor symptoms of the disease. Recordings were conducted no more than three hours after medication was taken. The healthy controls do not have symptoms associated with PD or any other neurological disease. Data for each PD participant is labeled with Movement Disorder Society-Unified Parkinson’s Disease Rating Scale Part III (MDS-UPDRS-III) [55], Hoehn & Yahr (H&Y) [56], and time post diagnosis. MDS-UPDRS-III is a clinician-scored monitored motor examination. Scores range from 0 to 132 points with scores below 32 points indicating mild motor impairment, scores from 33 to 58 points indicating moderate motor impairment, and scores of 59 or higher indicating severe motor impairment. The H&Y scale is a clinical tool used to describe the progression of PD. It ranges from Stage 1 (mild symptoms, minimal disability) to Stage 5 (severe disability, requiring full-time care). An H&Y score less than or equal to two corresponds to the disease being in the early stage. The time post diagnosis refers to the duration since each PD patient was diagnosed. Data for both the HC and PD groups are labeled with sex and age. The demographic and statistical details are summarized in Table 4.

**Table 4:** Patient demographics and calculated statistics derived from the dataset. μ: average, σ: standard deviation.

	PD Patients		HC Subjects	
	Male	Female	Male	Female
Number of subjects	25	25	25	25
Age [years] (μ±σ)	61.3 ± 11.4	60.7 ± 7.3	60.5 ± 11.6	61.4 ± 7.0
Range of age [years]	33–81	49–75	31–86	49–76
Time post diagnosis [years] (μ±σ)	8.7 ± 5.8	13.8 ± 12.4		
Range of time post diagnosis [years]	0.4–20	1–43		
MDS-UPDRS-III (μ±σ)	37.8 ± 22.1	37.6 ± 14.0		
Range of MDS-UPDRS-III	6–93	19–71		

### Ethics statement

The dataset used for this study is in the public domain and was provided to the authors de-identified. The dataset complies with the Helsinki Declaration. The collection of the dataset was approved by the Ethics Committee of the Clínica Noel, in Medellín, Colombia. Each participant signed a written informed consent.

### Computational approaches

The details of the implementation of the methods are shown in this section.

The code was written in Python using Google Colab.

The details of the Speech-to-Text Via Automated Speech Recognition step are as follows.

- Model: Whisper-1
- API Endpoint: https://api.openai.com/v1/audio/transcriptions
- Response_format parameter: set as “text”
- Other Parameters: none set or invoked

The details of the Text Embedding Via Large Language Model step are as follows.

OpenAI Endpoint Models:

- text-embedding-ada-002
  ○ API Endpoint: discontinued
  ○ Parameters: model = "text-embedding-ada-002"
- text-embedding-3-small
  ○ API Endpoint: https://api.openai.com/v1/embeddings
  ○ Parameters: model = "text-embedding-3-small"
  ○ dimensions = 768 (for text-embedding-3-small with reduced dimensions; otherwise, the parameter is not invoked, and the dimension is automatically set to the default maximum length).
- text-embedding-3-large
  ○ API Endpoint: https://api.openai.com/v1/embeddings
  ○ Parameters: model = "text-embedding-3-large"
  ○ dimensions = 768 (for text-embedding-3-large with reduced dimensions; otherwise, the parameter is not invoked, and the dimension is automatically set to the default maximum length).

Hugging face Transformers Endpoint Models: https://huggingface.co/docs/transformers/index

- BERT
  ○ Tokenizer: BertTokenizer.from_pretrained(’bert-base-uncased’)
  ○ Model: BertModel.from_pretrained(’bert-base-uncased’)
- XLNet
  ○ Tokenizer: XLNetTokenizer.from_pretrained(’xlnet-base-cased’)
  ○ Model: XLNetModel.from_pretrained(’xlnet-base-cased’)
- GPT-2
  ○ Tokenizer: GPT2Tokenizer.from_pretrained(’gpt2’)
  ○ Model: GPT2Model.from_pretrained(’gpt2’)

GENSIM Endpoint Models: https://radimrehurek.com/gensim/models/word2vec.html

- Word2Vec:
  ○ Model: glove-wiki-gigaword-300

The performance metrics, validation, state vector machine (SVM) classifier, and Support Vector Regression (SVR) are implemented with scikit-learn [67]
